# Prediction of the Drug–Drug Interaction Potential between Tegoprazan and Amoxicillin/Clarithromycin Using the Physiologically Based Pharmacokinetic and Pharmacodynamic Model

**DOI:** 10.3390/ph16030360

**Published:** 2023-02-26

**Authors:** Zhuodu Wei, Hyeon-Cheol Jeong, Min-Gul Kim, Kwang-Hee Shin

**Affiliations:** 1 College of Pharmacy, Research Institute of Pharmaceutical Sciences, Kyungpook National University, 80, Daehak-ro, Buk-gu, Daegu 41566, Republic of Korea; 2 Department of Pharmacology, Medical School, Jeonbuk National University, Jeonju 54907, Republic of Korea

**Keywords:** tegoprazan, clarithromycin, amoxicillin, PBPK, pharmacodynamics, drug interactions

## Abstract

Tegoprazan is a novel potassium-competitive acid blocker. This study investigated the effect of drug–drug interaction on the pharmacokinetics and pharmacodynamics of tegoprazan co-administered with amoxicillin and clarithromycin, the first-line therapy for the eradication of *Helicobacter pylori*, using physiologically based pharmacokinetic and pharmacodynamic (PBPK/PD) modeling. The previously reported tegoprazan PBPK/PD model was modified and applied. The clarithromycin PBPK model was developed based on the model provided by the SimCYP^®^ compound library. The amoxicillin model was constructed using the middle-out approach. All of the observed concentration–time profiles were covered well by the predicted profiles with the 5th and 95th percentiles. The mean ratios of predicted to observed PK parameters, including the area under the curve (AUC), maximum plasma drug concentration (C_max_), and clearance, were within the 30% intervals for the developed models. Two-fold ratios of predicted fold-changes of C_max_ and AUC from time 0 to 24 h to observed data were satisfied. The predicted PD endpoints, including median intragastric pH and percentage holding rate at pH above 4 or 6 on day 1 and day 7, were close to the corresponding observed data. This investigation allows evaluation of the effects of CYP3A4 perpetrators on tegoprazan PK and PD changes, thus providing clinicians with the rationale for co-administration dosing adjustment.

## 1. Introduction

Tegoprazan is a novel potassium-competitive acid blocker (P-CAB), reversibly and competitively inhibiting gastric H^+^/K^+^-ATPase. It can treat acid-related gastrointestinal diseases, including gastroesophageal reflux disease, gastric ulcers, and *Helicobacter pylori* (*H. pylori*) infection [1,2]. Compared with the traditional proton-pump inhibitors (PPIs), P-CAB shows a fast onset of action, reaching the immediate full potency from the first day of treatment; 4 to 5 days of daily dosing is required for PPIs to attain the full efficacy [3]. Furthermore, P-CAB has a relatively longer half-life than proton pump inhibitors and reversible inhibition of H^+^/K^+^ ATpase, resulting in higher night-time gastric acid suppression [4]. P-CABs have advantages over PPIs in inhibiting acid suppression and have been used as PPI alternatives in some treatment therapies [5,6,7,8,9]. For example, vonoprazan is more efficacious than PPIs in treating *H. pylori* [10]. Tegoprazan can be an effective PPI alternative in triple therapy of *H. pylori* eradication consisting of amoxicillin, clarithromycin, and PPIs [11,12,13].

Tegoprazan is a substrate of cytochrome P450 (CYP3A4) that metabolizes it into around 75% fraction of the major metabolite M1 (f_m_) [4]. Due to the high f_m_, drug–drug interactions (DDI) may occur between tegoprazan and CYP3A4 perpetrators, leading to significant alterations in tegoprazan plasma concentrations and the acid suppression effect. The clinical DDI studies demonstrated that the AUC and C_max_ of tegoprazan increased more than two-fold after co-administration of clarithromycin (CYP3A4 inhibitors) and amoxicillin (CYP2C8 inhibitors) compared to the administration of tegoprazan alone [11]. Furthermore, the DDI potential between tegoprazan and other agents affecting CYP3A4 (ketoconazole and rifampicin) has been predicted using a physiologically based pharmacokinetic (PBPK) modeling of tegoprazan [14].

PBPK modeling is a mathematical modeling incorporating the anatomical and physiological properties (e.g., organ volume, tissue flow rates, and tissue composition), physicochemical properties (e.g., lipophilicity, molecular, and acid dissociation constant) of a drug, study protocol, and formulation properties to predict the PK profiles of the investigated drug. Furthermore, PK and PD profiles can be evaluated simultaneously using the developed PBPK/PD model [15]. Assessing the DDI potential is one of the primary applications of the PBPK model [16,17,18]. Regulatory agencies, including the European Medicines Agency and the US Food and Drug Administration, have affirmed that some clinical trials can be replaced by the DDI simulation using PBPK modeling [19].

Two tegoprazan PBPK models have been built and applied recently [4,14]. The tegoprazan PBPK/PD model developed by Jeong et al. was a whole-body PBPK model; the model of major metabolite M1 was also included in the study [4]. Their final model was applied to simulate tegoprazan PK properties after administration of multiple doses, the postprandial PK profiles, and the stomach pH profiles. However, the PK and PD changes under DDI scenarios could not be predicted using their model. The tegoprazan minimal PBPK model developed by Yoon et al. consists of a single adjusted compartment and was verified by six clinical studies containing two DDI studies [14]. Their model was applied to simulate the DDI potential between tegoprazan and CYP3A4 perpetrators, including clarithromycin, ketoconazole, and rifampicin. However, the PD of tegoprazan after co-administration has not been reported.

Therefore, the aim of this study was to investigate the influence of DDI on the PK and PD of tegoprazan after co-administration of clarithromycin/amoxicillin using the tegoprazan PBPK/PD model.

## 2. Results

### 2.1. Clarithromycin Advanced Dissolution, Absorption, and Metabolism (ADAM) Model Development

**Figure 1:**
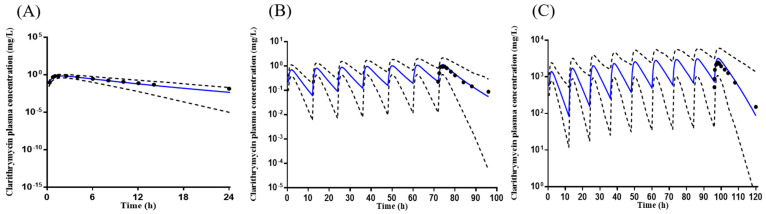
The mean observed (black dot) and predicted (solid blue line, *n* = 100) plasma clarithromycin concentration–time profiles. (**A**) After administration of a single 250 mg oral dose, observed *n* = 17 [20]; (**B**) After administration of multiple (7) doses (250 mg, every 12 h), observed *n* = 17 [20]. (**C**) After administration of amoxicillin 1000 mg/clarithromycin 500 mg for 5 days, observed *n* = 20 [11]. The black dotted lines represent the 5th and 95th percentiles. The observed data were adapted from references [11,20].

The initial model was optimized iteratively until the predicted PK profiles following the administration of single 250 mg clarithromycin adequately matched the observed data, and the predicted concentration–time profiles by the optimized model also fitted the PK profiles of clarithromycin after administration of multiple doses and co-administration of amoxicillin (Figure 1). Furthermore, the calculated mean ratios of predicted-to-observed C_max_ and AUC were within the 30% criteria (0.7–1.3), suggesting the developed clarithromycin ADAM had been successfully verified (Table S1, Supplementary Materials).

### 2.2. Development of Amoxicillin PBPK Model

**Figure 2:**
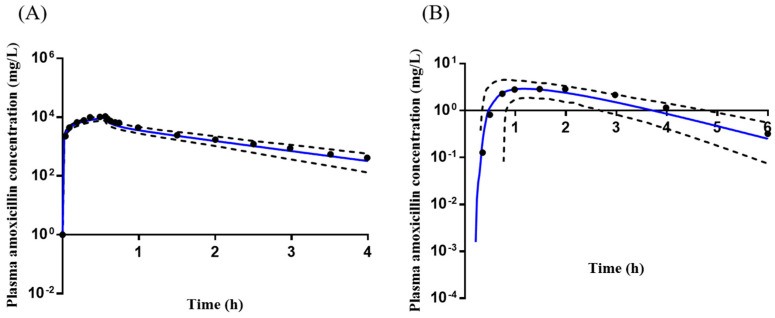
The mean observed (black dot) and predicted (solid blue line, *n* = 100) plasma concentration–time profiles of amoxicillin. (**A**) Administration of a single 250 mg capsule, observed *n* = 7 [23]; (**B**) administration of a single 259.6 mg tablet, observed *n* = 10 [23]. The black dotted and solid lines represent the 5th and 95th percentiles. The observed data were adapted from a reference [23].

The amoxicillin PBPK model was first constructed for intravenous (IV) dosing and then extended to oral dose. The predicted concentration–time profiles by the final IV and oral model are shown in Figure 2. The optimized models adequately captured the observations.

**Figure 3:**
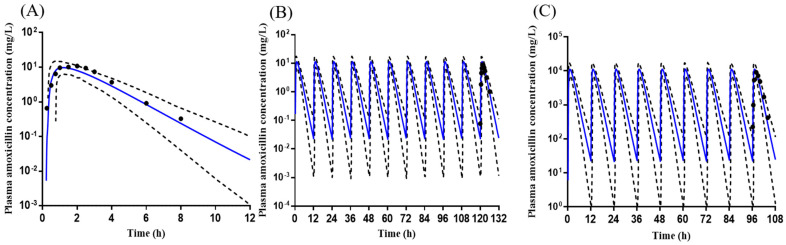
The mean observed (black dot) and predicted (solid blue line, *n* = 100) plasma concentration–time profiles of amoxicillin. (**A**) Administration of a single 875 mg amoxicillin tablet, observed *n* = 10 [21]; (**B**) administration of 1000 mg amoxicillin twice daily for 6 days, observed *n* = 15 [22]; (**C**) administration of 1000 mg amoxicillin twice daily for 5 days, observed *n* = 20 [11]. The solid gray lines represent the 5th and 95th percentiles. The observed data were adapted from references [11,21,22].

The optimized oral model was verified using three different clinical regimens [11,21,22]. The predicted concentration–time profiles with 5th and 95th percentiles covered all of the observed PK profiles of amoxicillin (Figure 3). In addition, the mean ratios of predicted-to-observed AUC, C_max_, and CL were within the 30% criteria (Table S2).

### 2.3. The Prediction of Tegoprazan PK and PD Changes under DDI Scenario

**Figure 4:**
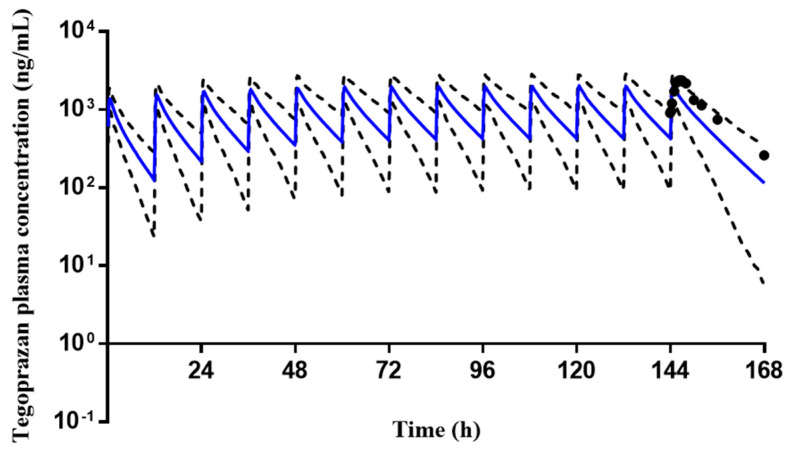
The mean observed (black dot, *n* = 20 [11]) and predicted plasma concentration–time profiles of tegoprazan 100 mg with amoxicillin 1000 mg and clarithromycin 500 mg twice daily for 7 days (solid blue line, *n* = 100). The gray dotted lines represent the 5th and 95th percentiles. The observed data were adapted from a reference [11].

**Figure 5:**
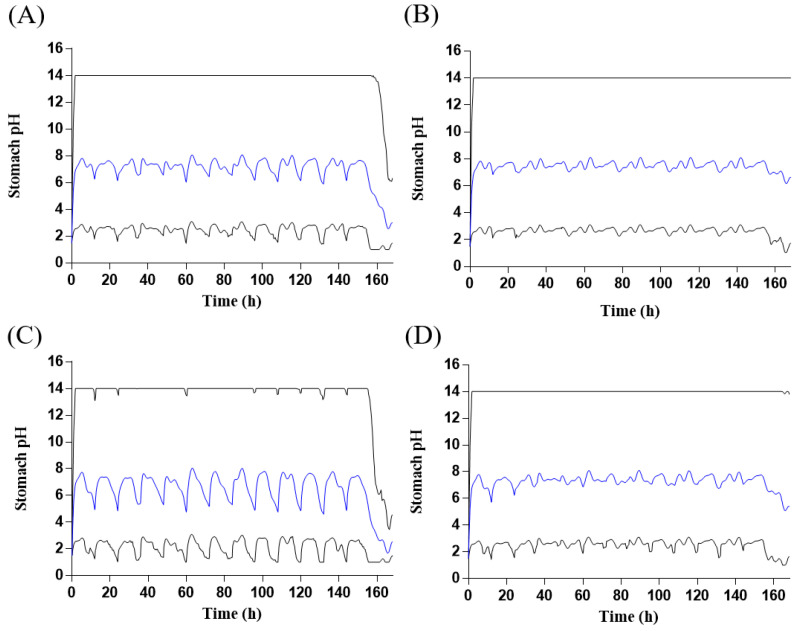
Predicted stomach pH–time profiles of (**A**) administration of 100 mg tegoprazan twice daily for 7 days; (**B**) administration of 100 mg tegoprazan with amoxicillin 1000 mg/clarithromycin 500 mg twice daily for 7 days; (**C**) administration of 50 mg tegoprazan twice daily for 7 days; (**D**) administration of 50 mg tegoprazan with amoxicillin 1000 mg/clarithromycin 500 mg twice daily for 7 days. Solid blue lines represent the predicted mean values; solid gray lines represent the predicted 5th and 95th percentiles.

The final tegoprazan PBPK model was applied to simulate the PK profile of tegoprazan after administration of tegoprazan 100 mg with amoxicillin 1000 mg and clarithromycin 500 mg twice daily (BID) for 7 days. The predicted PK profile covered the observed PK profile with the 5th and 95th percentiles (Figure 4). The results of predicted mean PK parameters, their ratios, and the mean predicted to observed DDI AUC_τ_ (the area under the concentration–time curve during a dosing interval at steady state) and DDI C_max_ ratios of tegoprazan with and without co-administration clarithromycin and amoxicillin are shown in Table 1. The mean ratios of predicted C_max_ and AUC_τ_ of tegoprazan to observations were 0.81 and 0.79, respectively, satisfying the 30% criteria. The DDI AUC_τ_ ratio and DDI C_max_ ratio were 0.91 and 0.65, respectively, within the less than two-fold criterion (0.5–1.2). Stomach pH–time profiles following two multiple dosages of 100 mg or 50 mg tegoprazan with amoxicillin 1000 mg/clarithromycin 500 mg BID for 7 days were predicted using the final tegoprazan PBPK/PD model (Figure 5). Predicted PD endpoints were calculated from the values for each of the key curves across the whole population. The calculated values were then compared with the observed data. All of the predicted values were close to the observed data (Table 2), indicating the great performance of the developed model.

## 3. Discussion

The influence of DDIs on tegoprazan PK and PD was investigated in this study using PBPK/PD modeling. The published tegoprazan PBPK/PD was refined and used as the substrate model. The PBPK model of clarithromycin with ADAM absorption model was constructed and verified using reported data from single, multiple and DDI scenarios. The amoxicillin PBPK model was developed from the IV model and then extended to the oral model. The final model could well describe the amoxicillin PK characteristics after single and multiple doses. The changes in tegoprazan PK profiles after co-administration with clarithromycin and amoxicillin were successfully described by comparing the mean predicted and observed tegoprazan concentration–time profiles, the mean ratios of PK parameters, the DDI AUC_τ_ ratio and DDI C_max_ ratio. Furthermore, the predicted PD endpoints containing median stomach pH and percent time with pH above 4 or 6 also fitted the observations well, indicating that the current model can simulate the PD effect of tegoprazan under the DDI scenario.

CYP3A4 mainly metabolizes tegoprazan in the liver with the f_m, CYP3A4_ of around 75% [4]. The systemic exposure of tegoprazan moderately increases after co-administration with amoxicillin and clarithromycin as a result of the CYP3A4 inhibition of clarithromycin [11]. The enzyme kinetic parameters of tegoprazan and interaction parameters of clarithromycin (Ki, _CYP3A4_, K_inact_) were reflected in the PBPK model, allowing the prediction of DDIs mechanistically. The fold-change ratios of C_max_ and AUC for the final model, in which the influence of clarithromycin and amoxicillin on tegoprazan PK were simultaneously considered, were within the two-fold criteria (0.65 and 0.91, respectively). That indicated that the final model could adequately simulate the PK alterations of tegoprazan caused by the clarithromycin inhibition of CYP3A. Although a clinical DDI study between tegoprazan and amoxicillin and clarithromycin has been reported, the doses of tegoprazan used in the clinical trials (100 mg every single dose) were not approved (50 mg every dose) [11]. In this case, the developed PBPK model could be applied to predict the PK interaction after administration of the approved dose. The predicted results could support the choice of appropriate drug therapy, a common application of the PBPK model.

PBPK modeling of tegoprazan was used previously to predict the tegoprazan DDI potential with CYP3A4 perpetrators such as clarithromycin, rifampicin, and ketoconazole [14], or to simulate the postprandial PK profiles and intragastric pH profiles after single or multiple dosing regimens [4]. In contrast with previous work, the stomach pH profiles and response parameters under DDI scenarios were first described in the present study. Moreover, the current model also considered the influence of amoxicillin on the alterations in tegoprazan PK and PD after co-administration with amoxicillin and clarithromycin. According to the in vitro stability study of tegoprazan using rCYPs by Jeong et al. [4], the intrinsic clearance rates of tegoprazan were estimated as 0.855, 0.614, 0.140 and 0.060 μL/min/pmol protein, respectively, in the presence of CYP3A4, CYP2C19, CYP2C9 and CYP2C8. This in vitro study showed that tegoprazan is a substrate of CYP2C8, although the intrinsic clearance of tegoprazan caused by CYP2C8 was not comparatively significant. The amoxicillin model developed in this study was first verified using both single and multiple dosing. Therefore, it could be used for further DDI predictions.

Although the DDI AUC_τ_ ratio and DDI C_max_ ratio satisfied the two-fold criteria, the mean predicted DDI C_max_ ratio was relatively lower than the observed DDI C_max_ ratio (Table 1). This phenomenon of underpredicted DDI C_max_ ratio was similar to the reported results of DDI simulation using the tegoprazan PBPK model [14]. The variability of observed data was considered as one possible reason due to the small number of clinical study participants (*n* = 20) [11]. Furthermore, the physiological factors and drug-specific factors such as the pH-dependent absorption of tegoprazan might significantly influence the C_max_ of tegoprazan after multiple dosing [4]. However, the value of AUC was used to reflect the magnitude of acid suppression of tegoprazan (P-CAB) instead of the value of C_max_ [24]. Therefore, the PD endpoints could not be significantly impacted by the lower DDI C_max_ ratio prediction.

The ability of the developed models to predict the PD response after co-administration was assessed by a visual check of PD endpoints. All of the predicted PD endpoints were in good agreement with the observed data. The stomach pH–time profiles of tegoprazan alone and tegoprazan with interactions were first compared in this study. By comparing the predicted stomach pH profiles between tegoprazan alone and co-administration (Figure 5), it could be seen that there were no significant changes in the stomach pH for both doses of tegoprazan, and even the mean AUC and C_max_ of tegoprazan increased more than two-fold after co-administration, indicating negligible clinical significance. This was consistent with the clinical report [11].

A limitation of this study is that, due to the lack of observed gastric pH profiles, the developed model could only be validated by comparing predicted PD endpoints with observed PD endpoints, not using gastric pH profiles. Once more data are available, the performance of the PBPK/PD model of tegoprazan could be more adequately verified. Another limitation is that the influences of tegoprazan on the PK and PD of clarithromycin and amoxicillin were not evaluated. Tegoprazan and clarithromycin are substrates of CYP3A, leading to a potential DDI by competitive enzyme inhibition. The increased mean concentration profiles of clarithromycin after co-administration of tegoprazan compared with the therapy without tegoprazan were reported [11]. We expect that further data on the interaction parameters, such as the values of concentration of inhibitor that supports half maximum inhibition and the inactivation rate of the enzyme of tegoprazan, can be obtained by in vivo or in vitro studies in the future. In that case, the influence of tegoprazan as an enzyme inhibitor might be investigated using the PBPK model.

## 4. Materials and Methods

### 4.1. Clarithromycin ADAM Model Development

The clarithromycin compound file with the first-order absorption model is available in the compound library of SimCYP^®^ simulator version 21 (Certara, NY, USA). To reflect the dynamic stomach pH after co-administration of tegoprazan with clarithromycin/amoxicillin, the ADAM module was applied in the absorption model of the clarithromycin PBPK model instead of the first-order absorption model. The initial parameters of the clarithromycin ADAM model were obtained from the SimCYP^®^ compound library, except for the absorption parameters. The final absorption parameters applied in this study included the default unbound fraction of drug in enterocytes, user input human jejunum effective permeability (P_eff, man_), and solubility–pH profile predicted by ChemAxon^®^.

The reported concentration–time profiles were extracted using Engauge Digitizer version 12.1. The clinical study after administration of single oral 250 mg amoxicillin [20] was used as the training dataset. Sensitivity analysis was used to pick out the parameters that had the greatest impact on the prediction results. Then, the sensitive parameters were optimized by parameter estimation in SimCYP^®^, based on the corresponding clinical data [25]. To conduct the parameter estimation, the option for objective function was set as weighted-least squares with weighting by reciprocal of observation squared, the option for minimization method was set as nelder-mead, and the maximum number of iterations was set to 20. Finally, the values of P_eff, man,_ and maximum rate of metabolism for CYP3A4 (V_max, CYP3A4_) were estimated using parameter estimation. The final input parameters are listed in Table 3.

The optimized model has been verified using a clinical study of clarithromycin after administration of 250 mg multiple (7) doses (every 12 h) [20], which is different from the study used for the optimization of the initial model. The virtual population and dosing regimen applied in SimCYP^®^ were consistent with the corresponding clinical data. The simulation population size for each study was set to 100 virtual persons (10 trials × 10 subjects in each trial). The predicted concentration–time profiles and the primary PK parameters containing the C_max_, the time required to reach the C_max_ (T_max_), and AUC_τ_ were compared between simulation results and observed clinical data.

### 4.2. Amoxicillin Model Development

An amoxicillin model has been reported to predict maternal and fetal drug exposures [26]. The model was not applied in the current study because the reported model has only been verified using a single dosing regimen. The multiple doses of amoxicillin used for DDI studies could not be adequately verified using the reported model (in-house data). Therefore, a whole new PBPK model of amoxicillin was constructed in this study using the middle-out approach. The amoxicillin model was initially constructed for IV dose and then extended to the oral route. A comprehensive literature review obtained amoxicillin’s physiological and PK parameters (absorption, distribution, metabolism, and excretion). The parameters not available in the literature were estimated using SimCYP^®^.

The clinical study of 250 mg IV dose [23] was applied to optimize the IV model. Once the IV dose-simulated concentration–time profiles matched the observed data, the IV model dose was extended to the oral dose by adding the absorption process input parameters. The first-order absorption model was chosen for amoxicillin. The mean first-order absorption rate constant (ka) and the fraction available from dosage form (fa) (1.08 and 0.9, respectively) were obtained from the literature [23]. The whole organ metabolic clearance was chosen for the elimination model. The renal clearance of amoxicillin used for the final model was 14.5, roughly accounting for 60% of the systemic clearance, consistent with the clinical report [27]. Finally, the oral model was optimized using the clinical study of a single 259.6 mg oral dose [23]. The final input parameters for the amoxicillin PBPK model are shown in Table 4.

The optimized amoxicillin model has been verified using one single 875 mg oral dose [21] and two multiple doses of 1000 mg BID for 6 or 5 days [11,28], different from the study used for the initial model development. The simulation conditions were set as the corresponding clinical data. The concentration–time profiles of each trial were simulated, and the primary PK parameters containing C_max_, T_max_, AUC, and clearance were compared between simulation results and observed clinical data.

### 4.3. Alterations in Tegoprazan PK and PD under the DDI Scenario

The tegoprazan PBPK model developed by [4] was applied in the present study without any adjustment of input parameters. The reported tegoprazan PBPK model was constructed using the physiochemical and PK characteristics of tegoprazan, including absorption (ADAM absorption model), distribution (full PBPK model), and elimination (enzyme kinetics model). This model has been successfully verified using single and multiple dosing regimens.

In predicting the DDI potential between tegoprazan and clarithromycin/amoxicillin, the tegoprazan PBPK/PD model was the substrate model (CYP3A4 victim). The clarithromycin ADAM model developed in this study was set as inhibitor 1 (CYP3A4 inhibitor), and the developed amoxicillin compound was set as inhibitor 2. The clinical study of repeated administrations of tegoprazan 100 mg with clarithromycin 500 mg and amoxicillin 1000 mg BID for 7 days [11] was used to verify tegoprazan DDI as CYP3A4 victim. The DDI simulations were generated according to the reported study. The simulated population size was 100 (10 trials × 10 subjects in each trial). The prediction performance was evaluated by comparing the simulated tegoprazan plasma concentration–time profiles, PK parameters, and the mean ratios of tegoprazan with and without co-administration of clarithromycin/amoxicillin. Two evaluation criteria were applied when assessing the DDI performance. The mean simulated C_max_ and AUC were within 30% of observed values, and the mean predicted to observed ratios of fold-changes of C_max_ and AUC were within the two-fold range criterion.

The PD model of tegoprazan applied in the current study was obtained from the tegoprazan PD model reported by Jeong et al. [4] with modifications. The indirect response sigmoid E_max_ model combined with the baseline stomach pH model was constructed in the reported model. The architecture of the developed PD model included one PD basic unit and two link units. The Lua scripts for baseline intragastric pH model and indirect response model are shown in Tables S3 and S4. The stomach pH range was restricted to 1 to 7.5 in their model, considering that stomach pH might be elevated due to the increased exposure to tegoprazan after DDI. The script for the range limit of stomach pH was deleted, leaving PD parameters unchanged. Then, the revised PBPK/PD model was applied to predict the stomach pH profiles and major PD endpoints (median stomach pH, percentage time with pH above 4 or 6 on day 1 and day 7 of treatment) for two different dosages: tegoprazan 50 mg or 100 mg BID co-administered with clarithromycin 500 mg and amoxicillin 1000 mg BID for 7 days. The predicted results were compared with the corresponding clinical study [11].

## 5. Conclusions

In conclusion, the influence of DDI on the PK and PD of tegoprazan after co-administration of clarithromycin and amoxicillin, the first-line therapy for eradication of *H. pylori*, was successfully simulated using the current tegoprazan PBPK/PD model coupled with the clarithromycin and amoxicillin PBPK model. The predicted alterations of C_max_ and AUC of tegoprazan were within the predefined criteria. Moreover, the PD profiles and endpoints of tegoprazan in the DDI study were first simulated using the tegoprazan PD model, and the predictions were consistent with the clinical study. Since the tegoprazan PBPK/PD model has been verified using reported DDI studies of tegoprazan for PK and PD, the current model could be utilized to anticipate changes in PK and PD of tegoprazan after co-administration with other CYP3A4-mediated agents. This may provide clinicians with a rationale for dosing adjustment of tegoprazan after co-administration with the drugs.

## Figures and Tables

**Table 1:** The observed and predicted mean C_max_, AUC_τ_, their ratios, and the fold change ratios for drug–drug interaction simulation.

DDI Prediction	C_max_ (ng/mL)			AUC_τ_ (ng × h/mL)			Fold Change Ratio					
	Pred. (*n* = 100)	Obs. (*n* = 20)	Ratio Pred./Obs.	Pred. (*n* = 100)	Obs. (*n* = 20)	Ratio Pred./Obs.	DDI C_max_ R			DDI AUCR		
							Pred.	Obs.	Pred./Obs.	Pred.	Obs.	Pred./Obs.
Tegoprazan alone	1283.5	1018.4	1.26	5326.5	5955.9	0.89						
Tegoprazan coadministration	1849.2	2285.6	0.81	12,652.0	16,045.0	0.79	1.46	2.24	0.65	2.45	2.69	0.91

Tegoprazan alone: tegoprazan 100 mg twice daily for 5 days. Tegoprazan coadministration: tegoprazan 100 mg with amoxicillin 1000 mg/clarithromycin 500 mg twice daily for 7 days. Observed data were obtained from Ghim et al. [11]. Data are expressed as geometric mean; AUC_τ_: area under the concentration–time curve from time zero to 24 h; C_max_: the maximum concentration; Pred.: predicted data; Obs.: observed data; C_max_ R: ratio of increased maximum plasma concentration; AUCR: ratio of increased area under the concentration–time curve from time zero to last sampling timepoint.

**Table 2:** The observed and predicted pharmacodynamic endpoints following two different administrations of tegoprazan with amoxicillin and clarithromycin.

Parameters	Tegoprazan 100 mg + Amoxicillin 1000 mg/Clarithromycin 500 mg Twice Daily for 7 Days		Tegoprazan 50 mg + Amoxicillin 1000 mg/Clarithromycin 500 mg Twice Daily for 7 Days	
	Observed [11] (*n* = 11)	Predicted (*n* = 100)	Observed [11] (*n* = 11)	Predicted (*n* = 100)
Median pH (min–max)	Day 1: 7.56 (7.31–8.45) Day 7: 7.39 (6.48–8.67)	Day 1: 7.82 (1.50–8.30) Day 7: 7.48 (6.25–8.40)	Day 1: 7.22 (6.48–8.33) Day 7: 6.91 (6.30–7.53)	Day 1: 7.49 (1.50–8.20) Day 7: 7.12 (4.77–8.25)
pH > 4 over 24 h (%)	Day 1: 97.38 Day 7:100.00	Day 1: 97.33 Day 7: 100.00	Day 1: 96.45 Day 7: 99.25	Day 1: 96.63 Day 7: 100.00
pH > 6 over 24 h (%)	Day 1: 96.97 Day 7: 99.42	Day 1: 94.50 Day 7: 100.00	Day 1: 90.11 Day 7: 88.13	Day 1: 93.83 Day 7: 81.21

min: minimum value; max, maximum value. Observed data were obtained from Ghim et al. [11].

**Table 3:** Final input parameters for clarithromycin PBPK model.

Parameters	Value	Source
Phys-chem properties		
Molecular weight (g/mol)	748	Simcyp compound library
LogP	1.7	Simcyp compound library
Compound type	Monoprotic base	
pKa	8.99	Simcyp compound library
B/P	1	Simcyp compound library
f_u_	0.18	Simcyp compound library
Absorption		
Absorption model	ADAM	
f_uGut_	1	Simcyp compound library
P_eff, man_ (×10^−4^ cm/s)	3.3	Parameter estimation
Solubility type	Solubility–pH profile	Predicted by ChemAxon^®^
Solubility (mg/mL)	453.66 (pH 6.8)	
	287.08 (pH 7.0)	
	92.78 (pH 7.5)	
	5.68 (pH 9.0)	
	2.96 (pH 11.0)	
	28.2 (pH 13.0)	
	295.67 (pH 13.5)	
Distribution		
Distribution model	Minimal PBPK	
V_ss_ (L/kg)	1.75	Simcyp compound library
Elimination		
Clearance type	Enzyme kinetics	
V_max, CYP3A4_ (pmol/min/pmol of isoform)	15.5	Parameter estimation
K_m, CYP3A4_ (μM)	22.3	Simcyp compound library
CL_R_ (L/h)	8.05	Simcyp compound library
Interaction		
Ki, _CYP3A4_ (μM)	10	Simcyp compound library
fu_mic_, _CYP3A4_	0.87	
K_app_ (μM)	12	
k_inact_ (1/h)	2.13	
Ki, _ABCB1 (P-gp)_ (μM)	4.0	
Ki, _SLCO1B1_ (μM)	0.35	
Ki, _SLCO1B3_ (μM)	0.7	

ADAM: advanced dissolution, absorption, and metabolism; LogP: octanol:water partition coefficient; pKa: acid dissociation constant; B/P: blood-to-plasma ratio; f_u_: unbound fraction; f_uGut_: unbound fraction of drug in enterocytes; f_a_: fraction available from dosage form; Ka: first-order absorption rate constant; P_eff, man_: human jejunum effective permeability; V_ss_: volume of distribution at steady state; V_max_: maximum rate of metabolism; K_m_: Michaelis–Menten constant; CL_R_: renal clearance; Ki: concentration of inhibitor that supports half maximum inhibition; fu_mic_: fraction of unbound drug in the in vitro microsomal incubation; K_app_: concentration of mechanism-based inhibitor associated with half maximal inactivation rate; k_inact_: inactivation rate of the enzyme.

**Table 4:** Final input parameters for amoxicillin PBPK model.

Parameters	Value	Source
Phys-chem properties		
Molecular weight (g/mol)	365.4	PubChem
LogP	0.9	PubChem
Compound type	Ampholyte	
pKa1	3.2	PubChem
pKa2	11.7	PubChem
B/P	0.55	Parameter estimation
f_u_	0.83	Drug bank
Absorption		
Absorption model	First order	
Fa	0.9	Zarowny et al. [23]
Ka	1.08	Zarowny et al. [23]
Distribution		
Distribution model	Full PBPK	
V_ss_ (L/kg)	0.31	Adjusted by Kp scalar
Kp Scalar	0.71	Parameter estimation
Prediction model	Method 2 (Rodgers and Rowland model)	
Elimination		
Clearance type	WOMC	
Hepatic CL_int_ (μL/min/10^6^)	0.3	Parameter estimation
CL_R_ (L/h)	14.5	Parameter estimation
Additional systemic CL (L/h)	7.5	Parameter estimation

LogP: octanol:water partition coefficient; pKa: acid dissociation constant; B/P: blood-to-plasma ratio; f_u_: unbound fraction; fa: fraction available from dosage form; ka: first-order absorption rate constant (1/h); V_ss_: volume of distribution at steady-state; WOMC: whole-organ metabolic clearance; CL_int_: intrinsic clearance; CL_R_: renal clearance.

## Data Availability

Data are contained within the article and Supplementary Material.

## Supplementary Materials

The following supporting information can be downloaded at: https://www.mdpi.com/article/10.3390/ph16030360/s1, Table S1: The observed and predicted mean PK parameters and the ratios of predicted to observed values by the final clarithromycin PBPK model; Table S2: The observed and predicted pharmacokinetic parameters and their mean ratios of amoxicillin by the final amoxicillin model; Table S3: The Lua script for baseline intragastric pH model; Table S4: The Lua script for indirect response model.
