# Supplementary material for: Prediction of the Drug–Drug Interaction Potential between Tegoprazan and Amoxicillin/Clarithromycin Using the Physiologically Based Pharmacokinetic and Pharmacodynamic Model
Source: Pharmaceuticals (Basel). 2023 Feb 26;16(3):360. doi: 10.3390/ph16030360 (PMC10054914; doi:10.3390/ph16030360)
Supplement: Supplementary file 1 [file pharmaceuticals-16-00360-s001.zip › pharmaceuticals-2200093-supplementary.pdf]

Table S1. The observed and predicted mean PK parameters and the ratios of predicted to observed values by the final clarithromycin PBPK model

Parameters	Observed (n = 17)	Predicted (n = 100)	Ratio (predicted/observed)
***Single 250 mg oral dose* [20]**			
$AUC_{0\text{-}inf}$ (mg×h/L)	4.36 (1.51)	3.97 (1.97)	0.91 (0.36–1.46)
C_{max} (mg/L)	0.78 (0.25)	0.71 (0.26)	0.91 (0.47–1.35)
T_{max} (h)	1.8 (0.7)	1.97 (0.27)	-
CL (L/h)	58.14 (16.8)	70.24 (33.01)	1.21 (0.54–1.87)
***Multiple doses of 250 mg, every 12 hours for seven dose* [20]**			
AUC_{τ} (mg×h/L)	6.86 (2.09)	7.72 (5.33)	1.13 (0.28–1.97)
C_{max} (mg/L)	1.14 (0.32)	1.15 (0.56)	1.01 (0.44–1.58)
T_{max} (h)	2.0 (1.1)	2.09 (0.30)	-
CL (L/h)	39.48 (11.16)	46.66 (37.74)	1.18 (0.17–2.19)

Observed and predicted PK parameters are presented as the arithmetic mean (SD); $AUC_{0\text{-}inf}$: area under the curve from zero to time infinity; AUC_{τ} : area under the plasma concentration-time curve during a dosing interval at steady state; C_{max}: the maximum concentration; T_{max}: the time of maximum drug concentration.

Table S2. The observed and predicted pharmacokinetic parameters and their mean ratios of amoxicillin by the final amoxicillin model.

Parameters	Observed	Predicted (n = 100)	Ratio (predicted/observed)
A Single 875 mg oral dose (n = 10) **[21]**			
$AUC_{0\text{-}inf}$ (ng×h/mL)	34180.0 (12940.0)	28378.0 (6760.2)	0.83 (0.46–1.20)
C_{max} (ng/mL)	12120.0 (4060.0)	10184.1 (2695.2)	0.84 (0.48–1.20)
T_{max} (h)	1.5 [1.0–3.0]	1.2 [0.7–2.0]	–
CL (L/h)	29.1 (11.0)	32.7 (8.2)	1.12 (0.61–1.63)
A multiple oral dose of 1000 mg twice daily for 6 days (n = 15) **[22]**			
AUC_{τ} (ng×h/mL)	27900.0 (8400.0)	32475.5 (7756.7)	1.16 (0.72–1.61)
C_{max} (ng/mL)	9370.0 (2090.0)	11648.8 (3075.1)	1.24 (0.81–1.67)
T_{max} (h)	1.5 [0.8–2.5]	1.2 [0.7–2.0]	–
CL (L/h)	38.6 (13.2)	32.6 (8.2)	0.85 (0.49–1.20)
Multiple oral doses of 1000 mg twice daily for 5 days (n = 20) **[11]**			
AUC_{τ} (ng×h/mL)	32594.5	31519.3	0.97
C_{max} (ng/mL)	9476.9	11250.6	1.19
T_{max} (h)	2.0 [1.0–4.0]	1.2 [0.7–2.0]	–
CL (L/h)	31.2	32.7	1.05

Observed and predicted PK parameters are presented as mean (SD), except for T_{max} : median [min–max]

for a single 875 mg oral dose; $AUC_{0\text{-}inf}$: area under the curve from zero to time infinity; AUC_{τ}: area under the plasma concentration-time curve during a dosing interval at steady state; C_{max}: the maximum concentration; CL: clearance.

Table S3. The Lua script for baseline intragastric pH model

```
function popSimSetup(...)
  sc:setNUserOdes(1)
end
function compoundSetup(...)
  sc:setIIVDistribution(1, sc.TRUNCATED_NORMAL_CV, -0.236, 0)    -- a1
  sc:setIIVDistribution(2, sc.TRUNCATED_NORMAL_CV, 0.05303, 0)   -- b1
  sc:setIIVDistribution(3, sc.TRUNCATED_NORMAL_CV, -0.5106, 0)   -- a2
  sc:setIIVDistribution(4, sc.TRUNCATED_NORMAL_CV, -0.1866, 0)   -- b2
  sc:setIIVDistribution(5, sc.TRUNCATED_NORMAL_CV, -0.1753, 0)   -- a3
  sc:setIIVDistribution(6, sc.TRUNCATED_NORMAL_CV, 0.4165, 0)   -- b3
  sc:setIIVDistribution(7, sc.TRUNCATED_NORMAL_CV, -0.3828, 0)  -- a4
  sc:setIIVDistribution(8, sc.TRUNCATED_NORMAL_CV, 0.01066, 0)  -- b4
  sc:setIIVDistribution(9, sc.TRUNCATED_NORMAL_CV, 0.359, 0)    -- a5
  sc:setIIVDistribution(10, sc.TRUNCATED_NORMAL_CV, -0.08818, 0) -- b5
  sc:setIIVDistribution(11, sc.TRUNCATED_NORMAL_CV, 0.1388, 0)   -- a6
  sc:setIIVDistribution(12, sc.TRUNCATED_NORMAL_CV, 0.4256, 0)   -- b6
  sc:setIIVDistribution(13, sc.TRUNCATED_NORMAL_CV, 0.2392, 0)   -- w
  sc:setIIVDistribution(14, sc.TRUNCATED_NORMAL_CV, 1.697, 0)    -- a0
end
function individualCompoundSetup(...)
  for i=1,14 do
     local P_indi = sc:sampleIIVDistribution(i)
     sc:setParameter(i,P_indi)
  end
end
function odeRateStep(t, xin, su, gu, P, ...)
  local a1, b1, a2, b2, a3, b3, a4, b4, a5, b5, a6, b6, w, a0
  a1     = P[1]
```

```
  b1    = P[2]
  a2    = P[3]
  b2    = P[4]
  a3    = P[5]
  b3    = P[6]
  a4    = P[7]
  b4    = P[8]
  a5    = P[9]
  b5    = P[10]
  a6    = P[11]
  b6    = P[12]
  w     = P[13]
  a0    = P[14]


  SUR = a0 + (a1*math.cos(t*w) + b1*math.sin(t*w)) + (a2*math.cos(2*t*w) + b2*math.sin(2*t*w)) + (a3*math.cos(3*t*w) + b3*math.sin(3*t*w)) + (a4*math.cos(4*t*w) + b4*math.sin(4*t*w)) + (a5*math.cos(5*t*w) + b5*math.sin(5*t*w)) + (a6*math.cos(6*t*w) + b6*math.sin(6*t*w))
  xout = xin + SUR
  return xout
end
```

Table S4. The Lua script for indirect response model

```
function popSimSetup(...)
   sc:setNUserOdes(1)
end
function compoundSetup(...)
   sc:setIIVDistribution(1, sc.TRUNCATED_NORMAL_CV, 0.71, 30)
   sc:setIIVDistribution(2, sc.TRUNCATED_NORMAL_CV, 1.32, 30)
end
function individualCompoundSetup(...)
   for i=1,7 do
      local P_indi = sc:sampleIIVDistribution(i)
      sc:setParameter(i,P_indi)
   end
end
function odeInitStep(xin, su, P, ...)
  local kin, kout
  kin = P[1]
  kout = P[2]
  su[1] = 1.5
  return su[1]
end
function odeRateStep(t, xin, su, gu, P, ...)
   local kin, kout
   kin = P[1]
   kout = P[2]
   gu[1] = kin * xin - kout * su[1]
     if su[1] > 14 then
        su[1] = 14
     elseif su[1] < 1 then
        su[1] = 1
     end
   return su[1], sc:feedbackIndivStomachpH(su[1])
end
```
